# Immune Cells Release MicroRNA-155 Enriched Extracellular Vesicles That Promote HIV-1 Infection

**DOI:** 10.3390/cells12030466

**Published:** 2023-01-31

**Authors:** Julien Boucher, Alyssa Rousseau, Catherine Boucher, Caroline Subra, Wilfried W. Bazié, Audrey Hubert, Emma Bourgeault, Abderrahim Benmoussa, Benjamin Goyer, Philippe A. Tessier, Caroline Gilbert

**Affiliations:** 1 Axe de Recherche Maladies Infectieuses et Immunitaires, Centre de Recherche du CHU de Québec-Université Laval, Québec, QC G1V 4G2, Canada; 2 U.S. Military HIV Research Program, Silver Spring, MD 20910, USA; 3 Henry M. Jackson Foundation for the Advancement of Military Medicine, Bethesda, MD 20817, USA; 4 Walter Reed Army Institute of Research, 503 Robert Grant Avenue, Silver Spring, MD 20910, USA; 5 Programme de Recherche sur les Maladies Infectieuses, Centre Muraz, Institut National de Santé Publique, Bobo-Dioulasso 01 BP 390, Burkina Faso; 6 Département de Microbiologie-Infectiologie et d’Immunologie, Faculté de Médecine, Université Laval, Québec, QC G1V 0A6, Canada

**Keywords:** HIV-1, micro-RNA, extracellular vesicles, pathogenesis, inflammation, immune activation, TRL7/8, calprotectin, miR-155

## Abstract

The hallmark of HIV-1 infection is the rapid dysregulation of immune functions. Recent investigations for biomarkers of such dysregulation in people living with HIV (PLWH) reveal a strong correlation between viral rebound and immune activation with an increased abundance of extracellular vesicles (EVs) enriched with microRNA-155. We propose that the activation of peripheral blood mononuclear cells (PBMCs) leads to an increased miR-155 expression and production of miR-155-rich extracellular vesicles (miR-155-rich EVs), which can exacerbate HIV-1 infection by promoting viral replication. PBMCs were incubated with either HIV-1 (NL4.3Balenv), a TLR-7/8 agonist, or TNF. EVs were harvested from the cell culture supernatant by differential centrifugation, and RT-qPCR quantified miR-155 in cells and derived EVs. The effect of miR-155-rich EVs on replication of HIV-1 in incubated PBMCs was then measured by viral RNA and DNA quantification. HIV-1, TLR7/8 agonist, and TNF each induced the release of miR-155-rich EVs by PBMCs. These miR-155-rich EVs increased viral replication in PBMCs infected in vitro. Infection with HIV-1 and inflammation promote the production of miR-155-rich EVs, enhancing viral replication. Such autocrine loops, therefore, could influence the course of HIV-1 infection by promoting viral replication.

## 1. Introduction

The immune system is rapidly dysregulated following human immunodeficiency virus (HIV) infection. The resulting dysfunction persists even when antiretroviral therapy (ART) lowers the viral load to undetectable levels [1]. Accordingly, understanding the early events that cause this immune dysfunction and identifying new therapeutic targets might lead to improved restoration of immune function in patients receiving ART. Recent studies indicate that extracellular vesicles (EVs), or their content, are biomarkers of pathological developments and comorbidities associated with HIV infection [2,3,4,5]. Since they can be recovered from plasma, they could prove helpful in non-invasive monitoring of the immune system status of PLWH.

In addition to being biomarkers, EVs have been shown to directly influence HIV infection by sustaining [6,7] or preventing [8,9] viral propagation in their capacities as agents of cell-to-cell communication and material exchange. During HIV infection, EV content is altered to include viral genetic material and viral proteins, such as *nef* [10,11], and these *nef*-enriched EVs have a pro-apoptotic effect on bystander CD4 T lymphocytes [12,13]. EVs secreted by infected cells also carry glycoprotein 120, and their presence in a viral preparation increases infectivity [14]. The element TAR, a viral RNA that can activate HIV transcription, has been found in EVs [6], as has the entire HIV genome [11]. TAR transferred by EVs appears to render naïve CD4 T lymphocytes more susceptible to infection [6]. HIV infection also appears to alter the host protein and genetic material contents into EVs. The disintegrin and metalloprotease ADAM17 are incorporated into EVs secreted by infected CD4TL in response to *nef* expression [13]. ADAM17 then activates TNF expression by recipient PBMCs, which increases viral replication in quiescent CD4TL [13]. EVs produced by infected CD4TL can modify other immune cell responses to the virus and thus influence disease outcome. For these reasons, analyzing EV content analysis during HIV-1 infection could provide valuable information on disease progression. 

Recent studies have shown that a microRNA, miR-155, becomes more abundant in plasmatic EVs during HIV-1 infection, and that this enrichment is strongly associated with immune activation [2,5]. MicroRNAs are approximately 22 nucleotides-long non-coding RNA [15]. They derive from a secondary hairpin structure in their microRNA precursor transcripts (pri-miRNA), which originate from either coding or non-coding regions of the human genome. Endoribonuclease DICER cleaves the hairpin structure to form the pre-miRNA, which is exported out of the nucleus to be processed by the protein complex DROSHA/DGCR8 to obtain two mature miRNAs [16]. Thereafter, these microRNA, respectively, associate with an argonaute protein to form RNA-induced silencing complexes whose function is to regulate gene expression at a post-transcriptional level [17]. The specificity of this regulation is bound to a two to eight nucleotide sequence called the seed region, then hybridizes with mRNA to inhibit translation or induce mRNA degradation [16,18].

The gene from which miR-155 originates is regulated by multiple transcription factors such as NF-κB and AP-1 [19]. Expressed mainly in hematopoietic cells [20], this gene is presumed to regulate hematopoiesis since its transfection into hematopoietic stem cells inhibits their differentiation into myeloid cells, megakaryocytes, or erythrocytes [21]. MiR-155 can target multiple genes that regulate many immune functions [22]. For example, it can downregulate Src homology-2 domain-containing inositol 5-phosphatase 1 (SHIP-1) mRNA in association with a myeloproliferative disorder in mice [23]. It can notably be pro-inflammatory, by inhibiting the expression of the suppressor of cytokine signaling 1 (SOCS1), thus promoting cytokine production [24].

Additionally, it is overexpressed in the PBMCs, CD4TL, and CD8TL of patients with HIV in comparison with uninfected individuals [25]. The same study reported a positive correlation between miR-155 levels in T lymphocytes and the expression of activation marker CD38 and senescence marker PD-1. Finally, miR-155 expression by CD8 T lymphocytes correlates positively with their differentiation into effector cells and memory effector cells and negatively with the proportion of naïve CD8TL [26]. Therefore, miR-155 might play a role in the cytotoxic response [26] and T lymphocyte activation during HIV infection [25]. 

Considering the high abundance of EV-borne miR-155 in PLWH [2] and its association with immune activation [5,27] and viral replication [28], we sought to understand its activity throughout HIV-1 infection. We found that large EVs released by HIV-1-infected PBMCs are enriched in miR-155, and miR-155-rich EVs potentiate HIV-1 infection by increasing virion production and integration of the viral genetic code into the DNA of infected cells.

## 2. Materials and Methods

### 2.1. Infection of Peripheral Blood Mononuclear Cells

Prior approval for this study was received from the CHU de Québec research ethics committee (CER-12-03-208 (2012–2021); CER-2019-4258). All participants provided written consent. Peripheral blood mononuclear cells (PBMCs) were isolated from healthy donors on a lymphocyte separation medium cushion [29]. PBMCs were washed and resuspended in HBSS (Hank’s balanced salt solution). Cell viability was determined by trypan blue coloration. For the 12-day experiment, PBMCs were distributed in 24-well plates at 2 × 10^6^/mL in RPMI 1640 containing exosomes-depleted 10% fetal bovine serum and 1% penicillin-streptomycin. The fetal bovine serum was exosomes-depleted by centrifugation at 100,000× *g* for 16 h. Cells were incubated with HIV-1 (100 ng of p24) (NL4.3BE) or with HIV-1 plus EVs enriched in miR-155 (NL4.3BE/EV-miR-155) for 2 h, then washed three times, resuspended in complete RPMI containing IL-2 (30 U/mL) with or without IL-15 (10 ng/mL), and cultured for 12 days at 37 °C under 5% CO2. The culture supernatant was harvested every 3 days, and cells were resuspended in Trizol reagent, in lysis buffer (Tris base 0.0075M, SDS 1%, EDTA 0.01M) for DNA extraction, or analyzed by flow cytometry. For the 48-h experiment, PBMCs were distributed in 24-well plates at 2 × 10^6^/mL in X-VIVO TM 15 medium, incubated with HIV-1 without or with EV enriched in miR-155 for 2 h, then washed three times, resuspended in X-VIVO TM 15, and cultured for 48 h at 37 °C under 5% CO_2_. The culture supernatants were harvested every 24 h, and cells were resuspended in Trizol reagent or lysis buffer (Tris base 7.5 mM, SDS 1%, EDTA 10 mM, for DNA extraction).

### 2.2. Peripheral Blood Mononuclear Cells Stimulation

PBMCs were distributed in 24-well plates at 2 × 10^6^ cells/mL in X-VIVO TM 15 medium, and incubated with R848 (1 µg/mL) or TNF (100 ng/mL) for 24 h at 37 °C under 5% CO_2_. The culture supernatant was saved for analysis, and the cells were resuspended in a Trizol reagent.

### 2.3. Infection Peripheral Blood Lymphocytes

Peripheral blood lymphocytes (PBL) were obtained from healthy donors by adhesion of PBMC to a cell culture flask for 3 h. Non-adherent cells were mitogen-stimulated with phytohemagglutinin-L (1 μg/mL) and then cultured for 3 days with IL-2 (50 U/mL) at a density of 2 × 10^6^/cells/mL in a complete RPMI medium at 37 °C under 5% CO_2_, then exposed to HIV-1 NL4.3BE (100 ng of p24) without or with EV-borne miR-155 (NL4.3BE/EV-miR-155) for 2 h. The cells were then washed and maintained at 2 × 10^6^ cells/mL in a complete RPMI culture medium containing IL-2 (30 U/mL). Every 3 days, the culture supernatants were saved, and cells were analyzed by flow cytometry.

### 2.4. CD4 T Lymphocytes

Human CD4TL from healthy donors were purified from PBMC using a Stem-sep human CD4 T cell enrichment kit according to the manufacturer’s instructions and cultured at 2 × 10^6^/mL in RPMI. Cells were mitogen-stimulated with phytohemagglutinin-L (1 μg/mL), cultured for 3 days with rhIL-2 (50 U/mL) then infected in a complete RPMI medium at a density of 1 × 10^7^/mL with purified HIV-1 NL4.3Balenv (20 ng of p24/10^6^ cells) or mock-infected for 2 h, then washed and maintained at 2 × 10^6^/mL in complete culture medium containing IL-2 (30 U/mL) with recombinant calprotectin [30] (5 μg/mL) or vehicle for 5 days. Complete RPMI 1640 culture medium contained FBS, penicillin G, streptomycin, glutamine (Wisent), primocin, and plasmocin from Invivogen (San Diego, CA, USA). In all culture media, bovine exosomes were eliminated from fetal bovine serum by O/N ultracentrifugation at 100,000× *g*.

### 2.5. Flow Cytometry

Washed and counted PBLs or PBMCs (5 × 10^6^ per experimental treatment) were incubated with 100 ng of FVS780 in HBSS for 30 min at 4 °C to stain dead cells. After centrifuging and resuspending in HBSS with 1% FBS, cells were incubated with human FC Block for 10 min at RT followed by the antibody cocktail for 45 min at RT (panels shown in Table S1), washed and resuspended in HBSS with 4% paraformaldehyde and held at 4 °C for 20 min to inactivate HIV-1, washed again, and analyzed with a BD FACSCanto™ II (Biosciences).

### 2.6. Purification of PBMC-derived EVs

Cell-free supernatants were incubated with 1 mM aldrithiol-2 (AT-2) for 24 h at 4 °C to inactivate HIV 1. Supernatants were centrifuged in 1.5 mL microtubes (Sarstedt product no. 72.692.210) in a ThermoScientific Sorvall microcentrifuge (no. 75-772-436) at 3000× *g* for 15 min, then for 30 min at 17,000× *g* to obtain two pellets (called 3K and 17K). The remaining supernatant was centrifuged in 1.5 mL microtubes (ThermoScientific no. 314352H01) in a ThermoScientific Sorvall WX100 Ultracentrifuge (no. 36-101-0807) with the F50L rotor at 100,000× *g* (acceleration = 9; deceleration = 0) for 60 min at 4 °C to obtain a third pellet (100K). All pellets were resuspended in 200 µL of microfiltered (0.22 µm) PBS 1X. One half was diluted in Trizol LS, and the other was used for DLS analysis. Both were stored at −80 °C.

### 2.7. Production of miR-155-Enriched EVs

Human embryonic kidney 293T cells (HEK293T) cells at a confluency of 30–50% were transfected with 5, 20, or 40 µg of a pMIG-155 plasmid using the calcium/phosphate method. The plasmid was diluted in a sterile 0.125 M CaCl2 solution, then mixed with HBS 2X. Transfection solution was added, and the cells were incubated for 16 h. The control condition received the transfection solution without plasmid. Cells were washed, then incubated for 24 h in DMEM containing 2% FBS. Cell-free supernatant was filtered 0.22 µm, then centrifuged in a ThermoScientific Sorvall WX100 ultracentrifuge (no. 36-101-0807) with the T1250 rotor at 100,000× *g* (acceleration = 9; deceleration = 0) for 60 min at 4 °C to pellet EVs.

### 2.8. Virus Preparation and Titration

NL4-3Balenv (R5) virus was produced in HEK293T by transient transfection, as described previously [31,32]. Transfected cells were washed and kept in a culture medium for 48 h. Cell-free supernatant was filtered at 0.22 µm, then centrifuged at 100,000× *g* (acceleration = 9; deceleration = 0) for 45 min in an Optima L-90K Beckman Coulter centrifuge with a 70 Ti rotor to pellet virus and EVs. For the co-production of NL4.3BE and EV-miR-155, HEK293T cells were transfected with both NL4.3BE plasmid and pMIG-155 plasmid. The rest of the procedure is exactly the same as described above. The viruses were titrated for all stock preparations and experiments using an in-house ELISA for the viral protein p24gag [33].

### 2.9. ELISA Calprotectin

Calprotectin (S100A8/A9) in the culture supernatant was measured using an in-house ELISA with detection based on the sandwich principle as previously described [34]. High-binding plates (96-wells) were coated overnight at 4 °C with 100 μL/well of antibody (polyclonal rabbit anti-human S100A9 diluted to 1.25 μg/mL in 0.1 M NaHCO3 buffer pH 9.6). The plates were washed three times with PBS/0.1% Tween 20, and blocked for 1 h at room temperature with 200 μL/well of this buffer supplemented with 2% BSA. Samples and standards (100 µL) were diluted in blocking buffer and deposited on the plates. Lysis buffer (PBS, 2.5% Triton X-100, 1% trypan blue, 0.05% Tween 20) was then added (25 µL), and the plates were incubated for 1 h at room temperature. After three washes with PBS/0.1% Tween 20, the plates were held for 1 h at room temperature with 100 μL/well of primary antibody (Hycult^®^ Biotech monoclonal mouse anti-human S100A8/A9 clone 27E10 diluted to 0.075 μg/mL in blocking buffer), washed three times, and contacted for 1 h with 100 μL/well of HRP-conjugated secondary antibody (goat anti-mouse IgG diluted 1/10,000 in blocking buffer). After three washes, 100 μL of TMB soluble colorimetric substrate for horse-radish peroxidase was added. The reaction was stopped by adding 100 μL of 0.18 M H2SO4. The optical density was read at 450 nm (ELX808, BIO-TEK Instruments, Winooski, VT, USA), and data were acquired and processed using KC4 software and a 4-parameter logistic, nonlinear regression model. The calprotectin standard was purified from cytosolic fractions of human neutrophils as described previously [35]. The detection limits ranged from 0.1 to 100 ng/mL.

### 2.10. ELISA IL-8

IL-8 in the culture supernatant was measured with a commercial ELISA kit from R&D systems. The manufacturer’s protocol was followed. The optical density was read at 450 nm (SpectraMax 190, Molecular Devices, San José, CA, USA).

### 2.11. Dynamic Light Scattering (DLS) analysis

EV samples were analyzed in spectrophotometer cuvettes using a Zetasizer Nano ZS (Malvern Instruments). Hydrodynamic diameter measurements were done in duplicate at room temperature.

### 2.12. RNA Extraction

RNA was extracted from cells (resuspended in Trizol^®^) and from their EVs (in suspension diluted in three volumes of Trizol^®^ LS) using the phenol/chloroform method [36]. Its concentration was measured using a BioDrop™ spectrophotometer. Its quality was considered suitable when the 260 nm/280 nm absorbance ratio was between 1.5 and 2. Total RNA were treated with DNase I (0.2 U/µL) in 10 µL of DNase buffer (10 mM Tris, pH 7.5; 2.5 mM MgCl2; 0.5 mM CaCl2) at 37 °C for 20 min. Then, DNase was inactivated at 65 °C for 10 min after adding 1 µL of EDTA 50 mM.

### 2.13. DNA Extraction

Genomic DNA was extracted from PBMCs by the phenol/chloroform method. The lysate was treated with proteinase K (1 mg/mL) for 5 min at 55 °C. Phenol/chloroform/isoamyl alcohol was added, followed by centrifugation at 12,000× *g* for 3 min. Chloroform was added to the aqueous phase, followed by centrifugation at 12,000× *g* for 3 min. DNA was precipitated from the second aqueous phase at −20 °C by adding absolute ethanol, 0.5 M NaCl, and GlycoBlue Coprecipitant (30 µg/mL final concentration), centrifugation at 12,000× *g* for 15 min. DNA concentration was measured with a BioDrop™ spectrophotometer. DNA quality was considered suitable for subsequent analysis when the 260 nm/280 nm absorbance ratio was between 1.8 and 2. DNA extract was diluted 10-fold for downstream analysis.

### 2.14. MicroRNA Quantification by RT-qPCR

Reverse transcription was achieved using the miScript RT kit. The 20 µL final volume contained 4 µL of miScript HiFlex 5X buffer, 2 µL of nucleic mix 10X, 2 µL of RNA-free water, and 2 µL of miScript RT mix combined with 10 µL of RNA (1 µg total RNA for cells and 100 ng for EVs). The reaction mixture was held at 37 °C for 60 min followed by 5 min at 95 °C in a GeneAmp^®^ PCR System 9700 (Applied Biosystems). Quantitative PCR was conducted in 96-well plates (Multiplate™, BioRad©) with miScript SyBr^®^ Green from Qiagen© using a CFX Connect™ Real-time system (Bio-Rad©). The qPCR cycling program was 15 min at 95 °C followed by 40 cycles of 15 s at 94 °C, 30 s at 55 °C, and 30 s at 70 °C. Standard curves for microRNA quantification were produced using synthetic microRNA in serial dilution over 10^8^ to 10^3^ copies.

### 2.15. mRNA RT-qPCR

Reverse transcription was achieved using a SuperScript IV Reverse Transcriptase kit on 5 µL of RNA according to the manufacturer’s instructions on a GeneAmp^®^ PCR System 9700 (Applied Biosystems). Quantitative PCR was performed on 1 µL of diluted DNA in a final volume of 10 µL using a QuantiTect SYBR Green PCR kit with the CFX Connect™ Real-time system (Bio-Rad©). The primer sequences are shown in Table S2. Fold change was calculated as 2^−ΔΔCt^ with PPIA as the housekeeping gene [37].

### 2.16. HIV-1 RNA Quantification

Total RNA extracted from 50 µL of plasma by the phenol/chloroform method as described above was resuspended in 15 µL of Tris/EDTA buffer. RT-PCR was performed on 5 µL of RNA with Superscript IV reverse transcriptase according to the manufacturer instructions on a GeneAmp^®^ PCR System 9700 (Applied Biosystems). The primer set targets the HIV-1 LTR region shown in Table S2 [38]. Reactions were performed with 1 µL of 5-fold diluted cDNA on a CFX384 Touch Real-Time PCR Detection System (Bio-Rad). The precision of our HIV RNA quantification method was validated with a quantification standard (NIH AIDS Reagent Program).

### 2.17. HIV DNA Standard Curve Production

We used ACH-2 cells as a quantification standard since they carry one copy of the integrated HIV genome per cell [39,40]. Cells were maintained in culture in accordance with NIH specifications, then centrifuged at 6000× *g* for 10 min. The pellets were resuspended in lysis buffer (10 mM Tris-HCl, 0.5% Tween 20-Triton X-100, 400 µg/mL proteinase K) at 10^7^ cells/mL, and incubated at 55 °C in a heating shaker for 16 h. Proteinase K was inactivated by holding at 100 °C for 10 min. The cell lysate was stored at −20 °C. A 10-fold serial dilution from 300,000 to 3 cells was used to generate our standard curve for total and integrated HIV DNA and CD3 gene quantification.

### 2.18. Quantification of HIV DNA

The preamplification and qPCR reactions for total and integrated HIV genome copies and CD3 gene quantification were performed as described previously with the same primer sets [41], but using DNA purified by the method described above. For all qPCR reactions, TaqMan™ Fast Advanced Master Mix (Invitrogen) was used. All preamplification reactions were performed on a GeneAmp^®^ PCR System 9700 (Applied Biosystems), and all qPCR reactions were performed on a CFX384 Touch Real-Time PCR Detection System (Bio-Rad).

### 2.19. Statistical Analysis

Statistical analyses were conducted using GraphPad Prism software version 8.2.1. with *p*-values below 0.05 considered statistically significant. Flow cytometry data were analyzed using FlowJo software version 10.6.1.

## 3. Results

### 3.1. HIV and Other Inflammatory Factors Induce miR-155 Expression in PBMCs

**Figure 1:**
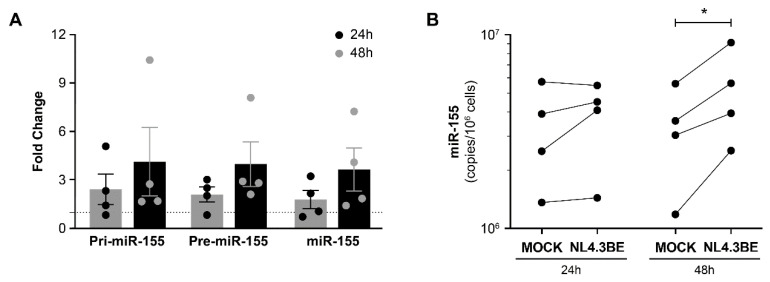
HIV increases precursor and mature miR-155 expression in PBMCs. PBMCs freshly isolated from HIV-negative donors (n = 4) were incubated with NL4.3Bal*env* virus (100 ng of p24 per 10^6^ cells) or diluent for 2 h, washed three times with PBS to eliminate free virus, then incubated for 24 h or 48 h. (**A**) Pri-miR-155, pre-miR-155, and miR-155 expression by HIV-infected PBMCs in comparison to their expression in mock-treated PBMCs, the latter being represented by the dotted line in the graph positioned at y = 1. Expression levels were measured by RT-qPCR. Fold change in the expression level was calculated as 2^−ΔΔCt^ with *PPIA* as the control gene. (**B**) Mature miR-155 concentration in PBMCs, calculated from a standard curve produced with synthetic miR-155. Each dot represents one donor. Significant difference (* *p* < 0.05) is based on parametric paired *t*-tests.

Our first objective was to assess the effect of HIV infection (with NL4.3BE particles) on the expression of mature miR-155 and its precursors (pri-miR-155 and pre-miR-155) in PBMCs, as measured by RT-qPCR. Precursors and mature miR-155 increased over the 48 h incubation period (Figure 1A). Overexpression of mature miR-155 was confirmed by the standard curve (Figure 1B). No significant modulation of miR-29a, miR-146a, or miR-223 expression was observed (Figure S1), suggesting that miR-155 overexpression by PBMCs might be a specific hallmark of HIV infection.

**Figure 2:**
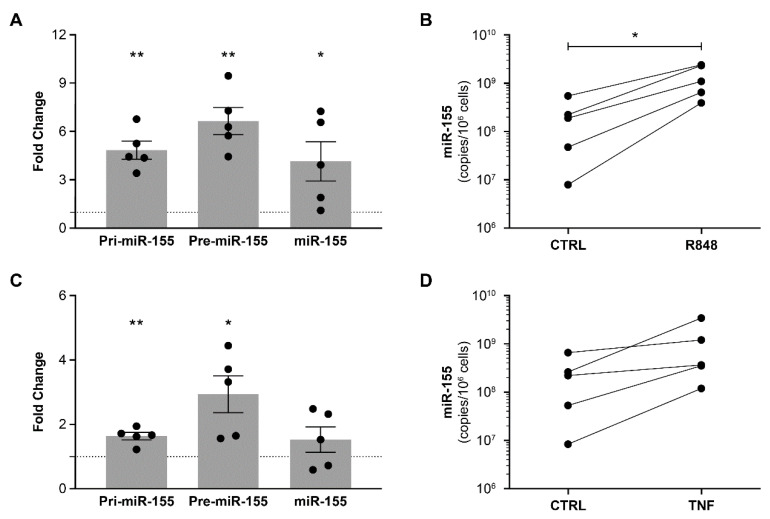
TLR7/8-agonist and TNF increase miR-155 expression in PBMCs. PBMCs freshly isolated from HIV-negative donors (n = 5) were incubated with (**A**,**B**) R848 (1 µg/mL) and (**C**,**D**) TNF (100 ng/mL) for 24 h. For the fold change graphs, pri-miR-155, pre-miR-155, and miR-155 expressions by activated PBMCs are compared to their respective expression in control PBMCs, the latter represented by the dotted line in the graph positioned at y = 1. Significant difference (* *p* < 0.05; ** *p* < 0.01) is based on parametric paired *t*-tests to compare the expression of a gene in activated PBMCs versus the expression of the same gene in control PBMCs. Expression levels were measured by RT-qPCR. Fold change in expression level was calculated as 2^−ΔΔCt^ with *PPIA* as the control gene. Concentrations were calculated from a standard curve produced using synthetic miR-155. Each dot represents one donor. Significant difference (* *p* < 0.05) is based on parametric paired *t*-tests.

Since other inflammatory factors could induce miR-155 expression, we examined the stimulation of PBMCs with R848, a TLR7/8 agonist known to activate the NF-κB and AP-1 transcription factors [42,43]. In addition, TLR7/8 recognizes and can be activated by HIV RNA [44]. Under these conditions, precursors pri-miR-155 and pre-miR-155 and mature miR-155 were all overexpressed within 24 h (Figure 2A). Absolute quantification of miR-155 by RT-qPCR validated the significant increase (Figure 2B). We also tested the effect of 24 h stimulation with TNF, a marker of systemic inflammation in HIV infection, and an activator of NF-κB and AP-1 [45,46]. TNF-stimulated PBMCs overexpressed pri-miR-155 and pre-miR-155, but not mature miR-155 (Figure 2C). Based on absolute quantification by RT-qPCR, the increase in miR-155, though noticeable, was not significant after only 24 h of stimulation (Figure 2D). The inflammatory environment associated with HIV infection thus appears to promote miR-155 expression by PBMCs.

Since calprotectin is produced by myeloid cells in response to inflammatory signals [47,48] and is a significant component of a specific protein signature in the genital tract of PLWH [49,50,51], we tested the effect of HIV, the TLR3 agonist poly(I:C), and the TLR7/8 agonist CLO97 on its production by monocyte-derived macrophages (MDM) stimulated for up to 4 days. HIV and CLO97 induced calprotectin production detectable by ELISA in MDM culture supernatant (Figure S2). Calprotectin is also an activator of the NF-κB transcription factor [35] and, therefore, could induce miR-155 expression by immune cells. Infected CD4 T lymphocytes were incubated for up to 5 days in the presence of calprotectin, and these results showed an increased pri-miR-155, pre-miR-155, and mature miR-155 (Figure S3A), confirmed by absolute quantification (Figure S3B). Furthermore, miR-155 was expressed preferentially compared to miR-29a, miR-29b, miR-92, and miR-223 (Figure S3C,D).

### 3.2. MiR-155 Overexpressing PBMCs Release miR-155 Enriched EVs

To determine the extent to which overexpressed miR-155 is released in EVs, we centrifuged HIV-pulsed PBMC culture supernatant sequentially at 3000× *g*, 17,000× *g*, and 100,000× *g* to obtain three pellets for EV size measurement by DLS. Size distributions in all three pellets differed little between the infected and the control conditions (Figure S4A). The expected 100–1000 nm hydrodynamic diameter EVs dominated the low-force (3K) and mid-force (17K) pellets, whereas the 20–200 nm diameter EVs were the most abundant in the high-force pellet (100K). DLS also provided a proxy measurement of the relative abundance of nanoparticles based on the number of photons scattered per second in the sample, expressed in thousands of counts per second (kcps), referring to the abundance of particles (Figure S4B). HIV infection was associated with a lower abundance of nanoparticles in the low-force pellet and in the mid-force pellet at 24 h. In the high-force pellet, no variation in nanoparticle abundance was observed. Overall, early HIV infection had a limited effect on the size and abundance of EVs released by PBMCs.

**Figure 3:**
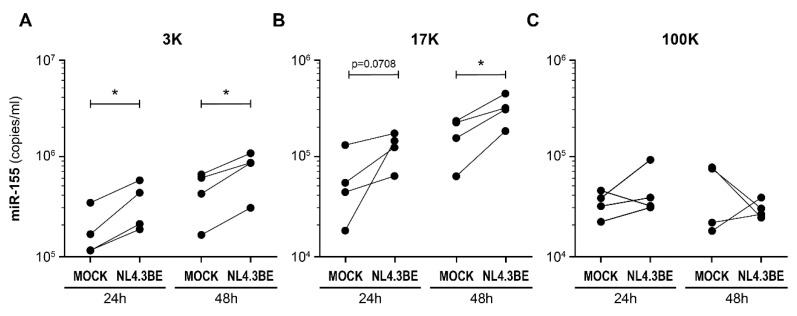
HIV-infected PBMCs release miR-155-rich large EVs. PBMCs freshly isolated from HIV-negative donors (n = 4) were incubated with NL4.3Bal*env* virus (100 ng of p24 per 10^6^ cells) or diluent for 2 h, washed three times with PBS to eliminate free virus, then incubated for 24 h or 48 h. Culture supernatants were incubated with 1 mM AT-2 to inactivate viral particles, then centrifuged sequentially at 3000× *g* (3K) (**A**), 17,000× *g* (17K) (**B**), and 100,000× *g* (100K) (**C**). Mature miR-155 in EVs was quantified by RT-qPCR, using a standard curve produced using synthetic miR-155. Each dot represents one donor. The significant difference (* *p* < 0.05) is based on parametric paired *t*-tests to compare the EVs derived from infected PBMCs versus the EVs derived from non-infected PBMCs (Mock).

Based on RT-qPCR, mature miR-155 was significantly more abundant in the low-force pellet after 24 h and 48 h of culture, and in the mid-force pellet at the 48 h time point (Figure 3). Enrichment in the latter was apparent but not significant at 24 h. The presence of miR-155 in the high-force pellet varied little (Figure 3).

**Figure 4:**
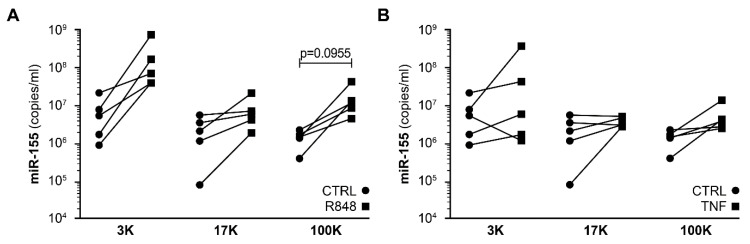
Stimulated PBMCs release miR-155-rich large EVs.PBMCs freshly isolated from HIV-negative donors (n = 5) were incubated with (**A**) R848 (1 µg/mL) and (**B**) TNF (100 ng/mL) for 24 h. Supernatants were centrifuged sequentially at 3000× *g* (3K), 17,000× *g* (17K) and 100,000× *g* (100K). Mature miR-155 was quantified by RT-qPCR, using a standard curve produced from synthetic miR-155. Each dot represents one donor. The significant difference is based on parametric paired *t*-tests to compare the activated condition (R848 or TNF) versus the control condition.

No increase in miR-29a, miR-146a, and miR-223 quantities was noted (Figure S5), and miR-155 stood out as the preferentially enriched miRNA in the low-force and mid-force centrifugal pellets. We noted that all four miRNAs were more abundant in the low-force pellet, while the high-force pellet contained the least, regardless of infection status (Figure S6). PBMCs stimulated with R848 released miR-155-enriched EVs mostly found within the low-force pellet (Figure 4A). PBMCs stimulated with TNF released EVs that were not enriched in miR-155 after 24 h (Figure 4B). These results show that miR-155-enriched EVs are released by PBMC stimulated by HIV or TLR7/8 agonists. Moreover, secreted miRNA appeared to be preferentially associated with larger EVs.

### 3.3. MiR-155-Enriched EVs Affect the Course of HIV-1 Infection

**Figure 5:**
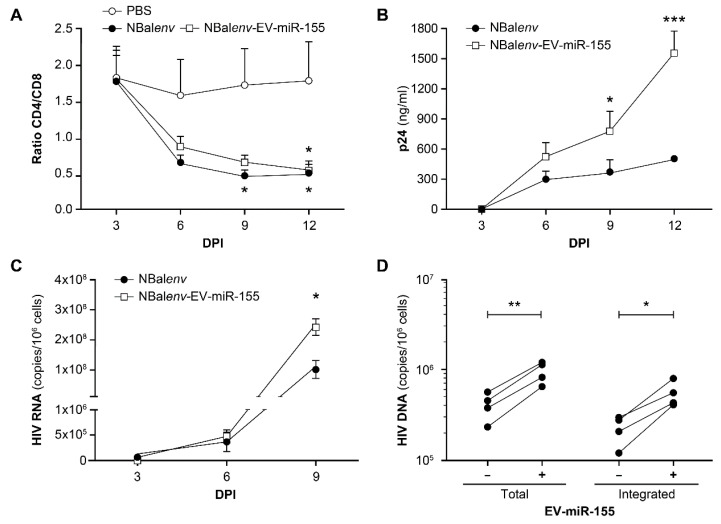
MiR-155 enriched EVs increase HIV-1 infection in vitro. PBMCs from different donors were pulsed with NL4.3BE or NL4.3BE/EV-miR-155, or mock-treated (with PBS) and cultured for 3, 6, 9, and 12 days. CD4+/CD8+ T cell ratio based on percentages determined by flow cytometry (**A**) Viral production based on quantitative ELISA (p24 capsid protein) (**B**) Viral load measured by RT-qPCR with a specific primer set targeting the viral LTR region (**C**) Values are averages (n = 4 independent donors). For A, B, and C, significant difference (* *p* < 0.05, *** *p* < 0.001) is based on a two-way ANOVA statistic test with the Bonferroni post-test. Total and integrated HIV DNA (based on a nested PCR assay) (**D**). The significant difference is based on a parametric paired *t*-test. DPI = days post-infection (* *p* < 0.05, ** *p* < 0.01).

Co-production of miR-155-enriched EV and NL4.3Balenv virus by transfected HEK293T cells (Figure S7A) shows that EV-miR-155 did not significantly change the infectivity of the virus on TZM-bl reporter cells (Figure S7B). In experiments on PBMC infection by NL4.3Balenv, the CD4/CD8 ratio was indifferent to the presence of EV-miR-155 (NL4.3BE/EV-miR-155). However, based on p24 measurement by ELISA and viral load measurement, the presence of the EVs significantly increased virus production (Figure 5). In a similar experiment with PBLs, the EVs again did not change the CD4/CD8 ratio (Figure S8A,B), but did not increase viral production (Figure S8C). In summary, miR-155-enriched EVs do not change the CD4/CD8 ratio in vitro, but increased viral production in PBMCs, but not in PBLs.

**Figure 6:**
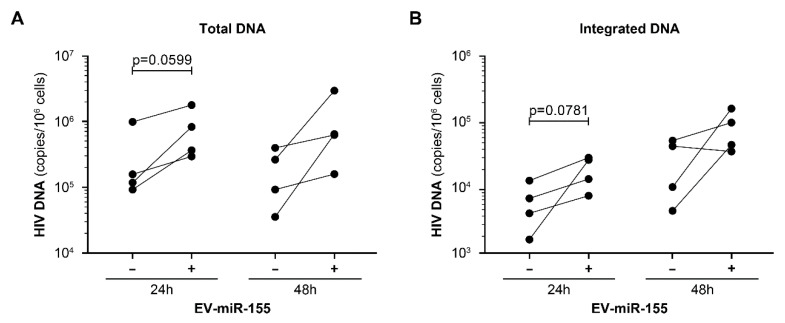
Vesicular miR-155 increases short-term HIV DNA production and integration. PBMCs from different donors were contacted with NL4.3BE or NL4.3BE/EV-miR-155 and cultured for 24 h and 48 h. Total (**A**) and integrated (**B**) HIV DNA was measured by a nested PCR assay. Each dot represents one donor. The significant difference for total and integrated HIV DNA measurement between PBMCs treated or non-treated at either 24 h or 48 h with miR-155-enriched EVs is based on a parametric paired *t*-test.

A crucial step in the HIV-1 replication cycle is the integration of the viral genome into the host genome [52]. To determine the effect of vesicular miR-155 on integration, integrated HIV DNA was measured by a nested qPCR that showed a significant increase in HIV DNA integration and total HIV DNA in the presence of EV-miR-155 (NL4.3BE/EV-miR-155) (Figure 6). Due to the integrated DNA was quantified at the end of the experiment, we needed to determine if the increase was a direct effect of the EVs or simply a consequence of a higher replication rate. By quantifying integrated DNA 24 h and 48 h after infection, we found that the effect of EV-miR-155 was more significant at 24 h (Figure 6), suggesting that vesicular miR-155 promotes HIV-1 integration sooner rather than later.

**Figure 7:**
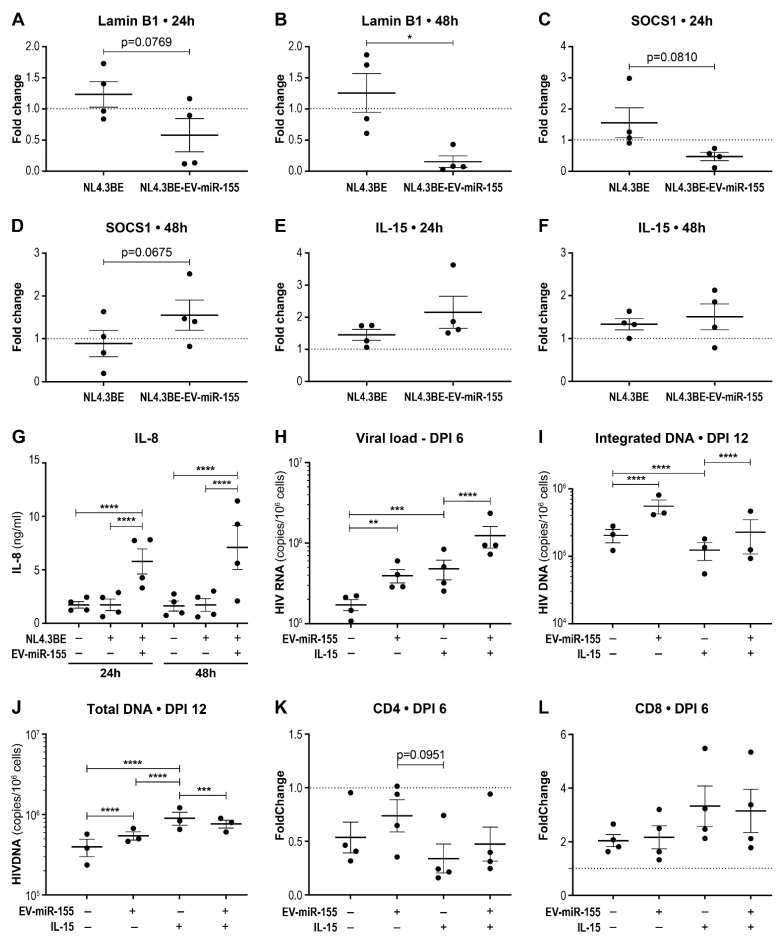
Vesicular miR-155 induces a pro-inflammatory environment that promotes viral replication. PBMCs from different donors were exposed to NL4.3BE or NL4.3BE/EV-miR-155, or mock-exposed, then cultured for 24 h and 48 h. SOCS1 (**A**,**B**), lamin B1 (**C**,**D**), and IL-15 (**E**,**F**) mRNA expression levels by the PBMCs were measured by RT-qPCR. PPIA was the housekeeping gene for every fold change calculation. The dotted line represents the baseline expression of the gene of interest by mock treated PBMCs. Significant difference was based on a parametric paired *t*-test (* *p* < 0.05). IL-8 was quantified by ELISA in the cell-free supernatant (**G**). Significant difference is based on two-way ANOVA with Tukey’s multiple comparisons test (**** *p* < 0.0001). PBMCs from different donors were exposed to NL4.3BE, NL4.3BE/EV-miR-155, IL-15 (10 ng/mL), or mock-exposed then cultured for 12 days. Viral load was measured by qRT-PCR at 6 days post-infection (DPI6) (**H**). Integrated and total HIV DNA was measured by a nested qPCR at DPI 12 (**I**,**J**). The mRNA expression of CD4 (**K**) and CD8 (**L**) was measured by RT-qPCR. Significant difference is based on two-way ANOVA with Tukey’s multiple comparisons test (** *p* < 0.01, *** *p* < 0.001, **** *p* < 0.0001).

To validate the biological activity of our EV-miR-155 preparations, we measured (by RT-qPCR) a significant downregulation of the relative expression of the well-described miR-155 targets lamin B1 and SOCS1 [24,53] in infected PBMCs (Figure 7A,B) (Figure S9). This suggests that vesicular miR-155 is transferred to recipient cells and is an effective down-regulator of its mRNA targets. Since SOCS1 suppression could be associated with a pro-inflammatory environment, we measured IL-15 and IL-8 expression in the PBMCs. IL-15 expression was increased in PBMCS that receives miR-155-rich EVs (Figure 7E,F). Quantification of extracellular IL-8 by ELISA showed that miR-155-rich EVs significantly increases IL-8 production by infected PBMCs (Figure 7G). When IL-15 was added to infected PBMCs, a higher replication rate was observed, reproducing the effect of miR-155-rich EVs (Figure 7H). Interestingly, when IL-15 and miR-155-rich EVs were added together, IL-15 boosted the effect of miR-155-rich EVs on the replication rate of HIV (Figure 7H). However, the addition of IL-15 resulted in a lower integration rate of viral DNA (Figure 7I). IL-15 was also associated with higher total HIV DNA when added alone, but not when added in combination with EV-miR-155 (Figure 7J). Next, we assessed the effect of IL-15 on CD4 and CD8 levels by RT-qPCR [54]. As expected, HIV infection was generally associated with a lower CD4 level and a higher CD8 level (Figure 7K,L). The addition of IL-15 was associated with a reduction in CD4 and an enhancement of CD8 expression.

## 4. Discussion

Studies during the past decade have revealed that miR-155 is present at elevated concentrations in plasmatic EVs of PLWH, and appears to be a biomarker of immune activation and viral replication in patients receiving antiretroviral therapy [5,27,28]. In the present study, we aimed to identify the factors that induce its overexpression and their impact on HIV-1 infection. HIV-1 appeared to increase pri-miR-155 expression, which is the primary RNA transcript of the miR-155 host gene, and pre-miR-155 expression, which is the hairpin structure excised from the pri-miR-155 that contains the miR-155 sequence. This leads to the overexpression of mature miR-155, as does TLR7/8 activation by R848, which mimics activation by viral RNA. HIV-1 infection is also associated with persistent elevated TNF [46], a known activator of miR-155 expression in endothelial cells [55,56] and in synoviocytes [57], which is consistent with our PBMC results. In addition, considering that calprotectin has a dominant presence in the genital epithelium and that this protein complex contributes to the generation of an autocrine activation loop of the inflammatory cascade (Figure S2), it was relevant to verify the effect of calprotectin on miR-155 production in the context of HIV infection. As expected, the results showed that calprotectin increases the expression of miR-155 and its precursor forms.

MiR-155 host gene transcription is upregulated by a variety of factors R848 [58] and TNF [55,56,57] in addition to infection by HIV-1 [59]. Therefore, we proposed that miR-155 upregulation in response to these factors occurs via NF- κB activation [19]. The expression of miR-146a and miR-223 are also regulated by the transcription factor NF-κB [60,61]. However, calprotectin and HIV had a negligible effect on the expression of both micro-RNAs (Figures S1 and S3). These results suggest that miR-155 regulation in PBMCs, independently or not of NF κB effects, is mainly expressed in inflammatory and infectious conditions.

EVs transport proteins and genetic material and participate in intercellular communication [62]. EVs content may be modified in response to cellular stress [63,64]. Therefore, we expected the miRNA content of EVs to reflect miRNA overexpression in cells. This was validated for miR-29a, miR-146a, miR-155, and miR-223 in response to infection (Figure 3, Figure 4, and Figure S5). In addition, these four miRNAs were mostly associated with larger EVs (obtained by centrifuging at 3000× *g* and 17,000× *g*). Significant upregulation of miR-155 in cells and EVs strengthen previous observations showing that large EV-miR-155 in plasma of PLWH are robust biomarkers of viral rebound [28] and immune activation [27].

It has been shown that miR-155 transfection into J-Lat 5A8 cells limits HIV-1 reactivation by inhibiting TRIM32 expression [65], and that transfection into ME-180 cells inhibits HIV-1 replication by suppressing TGF-β signaling [66]. The differences between these findings and ours could be attributed to using the more relevant model of primary cells. Whether miRNA transferred from EVs can significantly impact recipient cells has been controversial. One group has even suggested that vesicular miRNA is rarely transferred to neighboring cells, and that such transfers are biologically irrelevant [67]. However, others have shown in vivo that dendritic-cell-derived vesicular miR-155 is transferred to myeloid cells and lymphocytes and can down-regulate SHIP-1 in tissues and increase cytokine production in response to LPS [68], suggesting that vesicular miR-155 is biologically relevant, at least to the immune response. Another team has observed that vesicular miR-155 transfer leads to inhibition of enterovirus A71 replication in the nerve cell line SK-N-SH [69]. These results show that the EV-mediated transfer of miR-155 can modulate viral replication in recipient cells. This study strengthened our preliminary in vivo observations in humanized mice experiments [70]. In conclusion, our results reveal that our EV-miR-155 preparation is functional and influence the course of HIV-1 infection in primary cells by increasing the virus genome integration. To gain a better characterizing of our heterogeneous EV preparations, the iodixanol velocity gradient that allows an optimal separation of EVs and HIV 1 particles could be used to determine which EV subtype has a greater role in HIV-1 infection and to decipher their mechanism of action [71].

Here, we validated that the vesicular transfers of miR-155 to cells are biologically relevant by measuring SOCS1 downregulation in PBMCs, thus offering insight into the mechanism by which miR-155 could enhance HIV-1 replication. Reportedly, MiR-155 can suppress SOCS1 expression by binding to mRNA [24]. SOCS1 suppression has been associated with greater immune activation in HIV-1-infected patients [72]. SOCS1 inhibits the JAK-STAT signaling pathway, which induces cell activation in response to cytokines [24,73]. Considering that activated cells have a higher viral production rate [74] and that miR-155-rich EVs can suppress SOCS1 expression in bystander PBMCs, we suspect that suppressed expression of SOCS1 in bystander cells promotes an activated state and accelerates viral replication. Another clue comes from the inhibition of lamin B1 in PBMCs treated with EV-miR-155. Neutrophil-derived EV-miR-155 has been shown to diminish lamin B1 expression in intestinal epithelial cells [53]. Lamin B1 is a nuclear membrane protein, and its downregulation leads to nuclear instability and slowed repair of damaged DNA [53]. Such instability could facilitate access of viral DNA to the host DNA and explain how miR-155 leads to more integration of the HIV genome. Others have shown that miR-155 activates NF-κB, thereby inhibiting B-cell lymphoma 6 [75,76,77], and can increase inflammatory responses in endothelial cells [77].

The presence of miR-155-rich EVs was associated with elevated IL-8 and IL-15 expression. It has been recently reported that miR-155 upregulates IL-8 production via a SOCS1 downregulation [78]. To our knowledge, this is the first time that miR-155 is associated with increased IL-15 expression. In a humanized mice model, miR-155 knock-out resulted in an IL-15 downregulation [79]. Our results showed that IL-15 favored HIV replication. It has been previously reported that plasmatic IL-15 strongly correlates with a higher viral load in PLWH [80]. IL-15 also increases the susceptibility of CD4TL to HIV-1 infection [81]. We showed that IL-15 boosted the effect of EV-miR-155 to further increase viral replication. IL-15 signaling goes through the JAK-STAT pathway, and its signal can be suppressed by SOCS1 [82]. Since EV-miR-155 downregulated SOCS1 expression, it is possible that miR-155 amplify the effect of IL-15 on viral replication by downregulating SOCS1 in target cells. IL-15 is mostly produced in myeloid cells [83]. It is notable that EV-miR-155 had a negligible effect on viral replication in PBLs (i.e., without myeloid cells). This suggest that the mechanism by which EV-miR-155 promote HIV-1 infection is likely IL-15-dependant.

We also showed that IL-15 was associated with a lower quantification of HIV-1 integrated DNA. IL-15 is known to increase the anti-HIV-1 effect of CD8TL and NK cells [84,85]. Since the integrated DNA was quantified later in the experiment, we suspected that CD8 activity was primed by IL-15, which lead to a partial clearing of the infected cells. CD8 levels were effectively increased, and CD4 levels decreased in the presence of IL-15.

In conclusion, miR-155 expression increases in PBMCs infected with HIV-1 and exposed to inflammatory factors. These results in the secretion of miR-155 carried by large EVs suggest these EVs are more than biomarkers of immune dysfunction and viral rebound in HIV-1 infection; these EVs are functional biomarkers of both phenomena. We have shown that EV-miR-155 directly increases viral replication and promotes HIV-1 DNA integration during the early stages of infection. The mechanism by which miR-155 enhances HIV replication still needs further investigation, and its comprehension could unveil new potential targets for HIV-1 treatments.

## Data Availability

The study protocol, results, and informed consent documents will be made available to researchers upon request from the corresponding author. Researchers will be asked to complete a concept sheet for their proposed analyses to be reviewed, and the investigators will consider the overlap of the proposed project with active or planned analyses, and the appropriateness of the study data for the proposed analysis.

## Supplementary Materials

The following supporting information can be downloaded at: https://www.mdpi.com/article/10.3390/cells12030466/s1, Figure S1: MiR-155 is preferentially overexpressed by HIV-infected PBMCs; Figure S2: Calprotectin secretion by monocyte-derived macrophages; Figure S3: Calprotectin enhances miR-155 expression in CD4TL infected with HIV-1; Figure S4: HIV infection has little effect on the size and relative abundance of EVs released by PBMCs; Figure S5: MiR-155 is preferentially enriched in EVs secreted by HIV-infected PBMCs; Figure S6: EV-borne miRNAs are associated mostly with larger EVs; Figure S7: NL4.3BE and EV-miR-155 co-production by HEK293T cells; Figure S8: Impact of EV-miR-155 on infection of PBL cells by HIV-1; Figure S9: EV-miR-155 downregulates SOCS1 mRNA independently of the infection status; Table S1: Antibody panels used for flow cytometry analysis; Table S2. Primer sets used for PCR.
