# Supplementary material for: Immune Cells Release MicroRNA-155 Enriched Extracellular Vesicles That Promote HIV-1 Infection
Source: Cells. 2023 Jan 31;12(3):466. doi: 10.3390/cells12030466 (PMC9914104; doi:10.3390/cells12030466)
Supplement: Supplementary file 1 [file cells-12-00466-s001.zip › cells-2090104-supplementary.pdf]

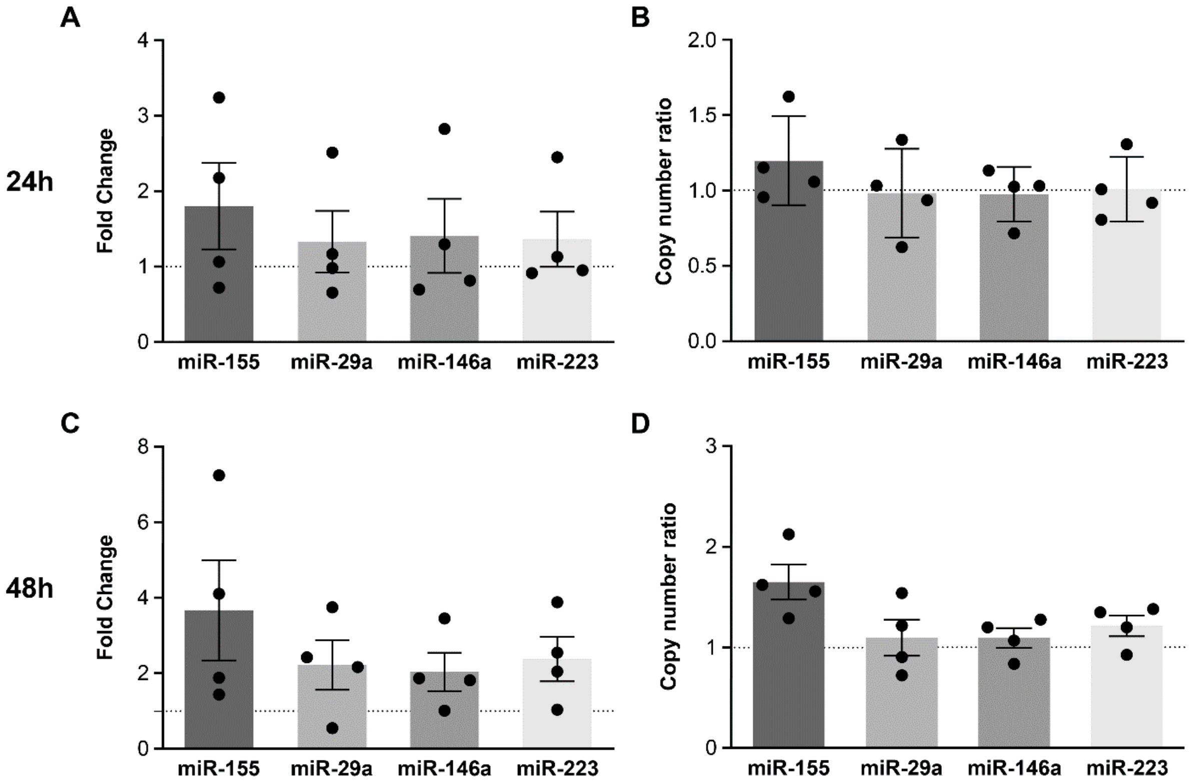

Figure S1. MiR-155 is preferentially overexpressed by HIV-infected PBMCs.
PBMCs freshly isolated from HIV-negative donors (n = 4) were incubated with NL4.3Bal*env* virus (100 ng of p24 per 10^6 cells) or diluent for 2h, washed three times with PBS to eliminate free virus then incubated for 24h or 48h. Mature miRNA was quantified by RT-qPCR. Fold change was calculated as $2^{-\Delta\Delta Ct}$ and *PPIA* was the housekeeping gene to compare miRNA expression in HIV-infected PBMCs versus mock-treated PBMCs. The absolute concentration in EVs was calculated from a standard curve obtained using synthetic miRNA. The copy number ratio was the number of miRNA copies in NL4.3Bal*env* samples divided by the number in control samples. The dotted line represents baseline expression. Each dot represents one donor. Significant difference is based on two-way ANOVA with Tukey's multiple comparisons test.

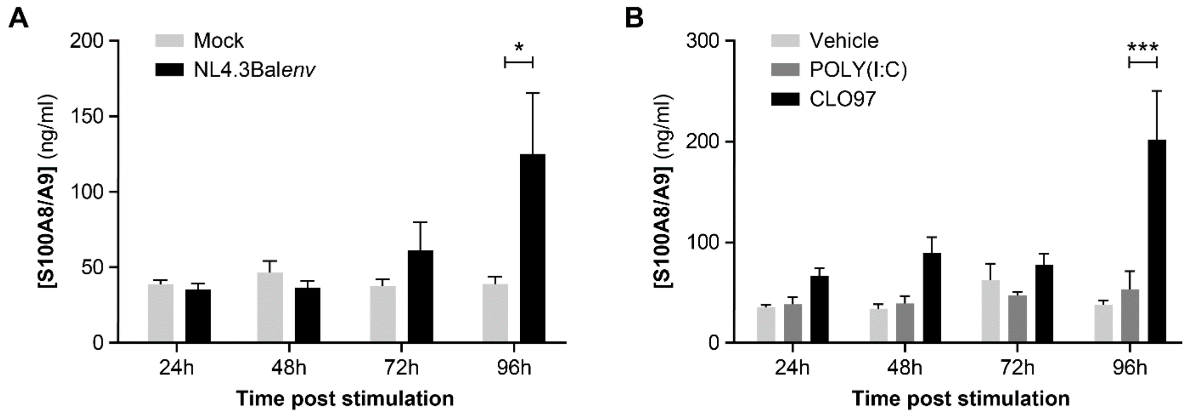

Figure S2. Calprotectin secretion by monocyte-derived macrophages
Monocyte-derived macrophages cells were exposed to NL4-3*Balenv* (10 ng p24 per 100,000 cells) or mock-exposed (A) or stimulated with agonists poly(I:C) 2.5 µg/mL or CLO97 20 µmol/L (B). Calprotectin in the supernatant was quantified by ELISA (minimally in triplicate) every 24h. Graphed values are mean ± SEM of 3 independent experiments. NL4-3*Balenv* comparison to the control (mock) condition is based on a paired T-test. Agonist comparison is based on two-way ANOVA (*p < 0.05, ***p < 0.001).

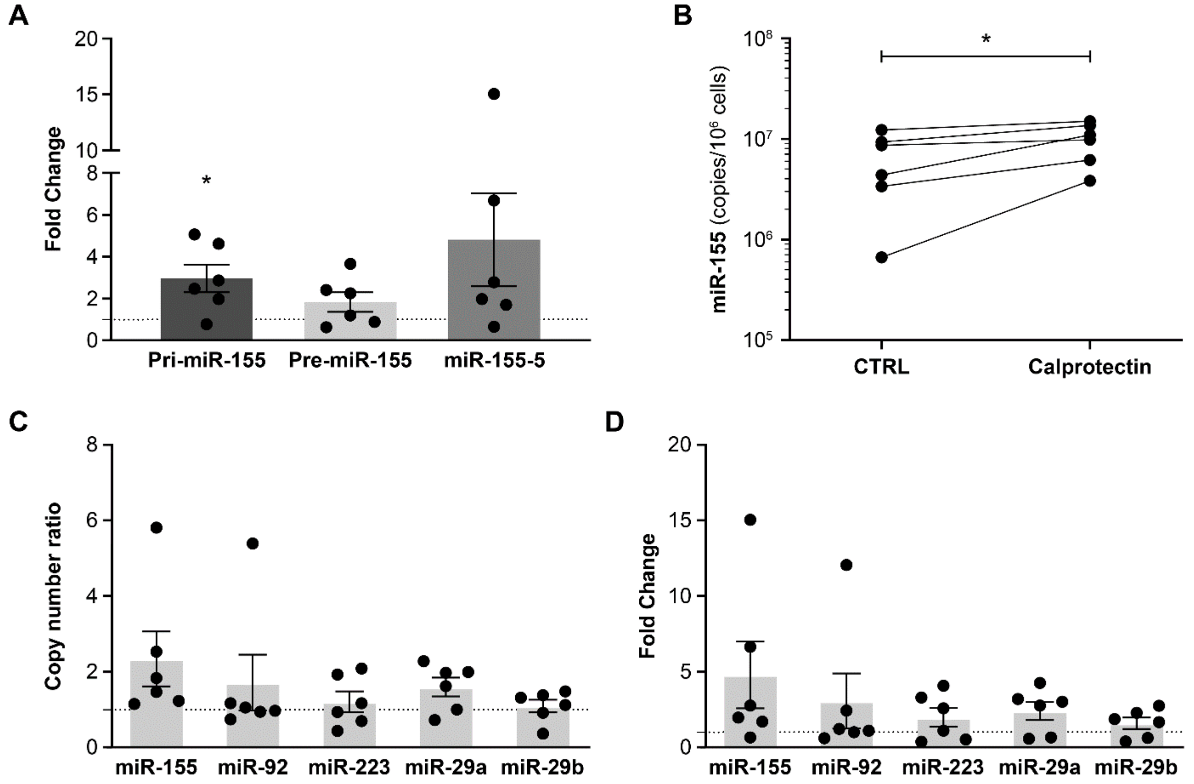

Figure S3. Calprotectin enhances miR-155 expression in CD4TL infected with HIV-1. CD4TL from peripheral blood from HIV-negative donors (n = 6) were incubated with NL4.3Bal*env* virus (100 ng of p24 per 10^6 cells) or diluent for 2h, washed three times with PBS to eliminate free virus then incubated with calprotectin (10 µg/mL) for 5 days. For the fold change and copy number ratio graphs, pri-miR-155, pre-miR-155 and miR-155 expression by activated PBMCs are compared to their respective expression in control PBMCs, the latter being represented by the dotted line in the graph positioned at y=1. Significant difference (*p < 0.05) is based on parametric paired t-tests to compare the expression of a gene in activated PBMCs versus the expression of the same gene in control PBMCs. **A.** Pri-miR-155, pre-miR-155, and miR-155 and expression measured by RT-qPCR. Fold change was calculated as $2^{-\Delta\Delta Ct}$ and *PPIA* was the housekeeping gene. The dotted line represents baseline expression. **B.** Mature miR-155 concentration in PBMCs was calculated from a standard curve produced using synthetic miR-155. Significant difference (*p < 0.05) is based on parametric paired t-tests. **C and D.** Comparison of the expression of 5 miRNAs by calprotectin-activated PBMCs, repesented by fold change and copy number ratio. The copy number ratio was the number of miRNA copies in NL4.3Bal*env* samples divided by the number in control samples.

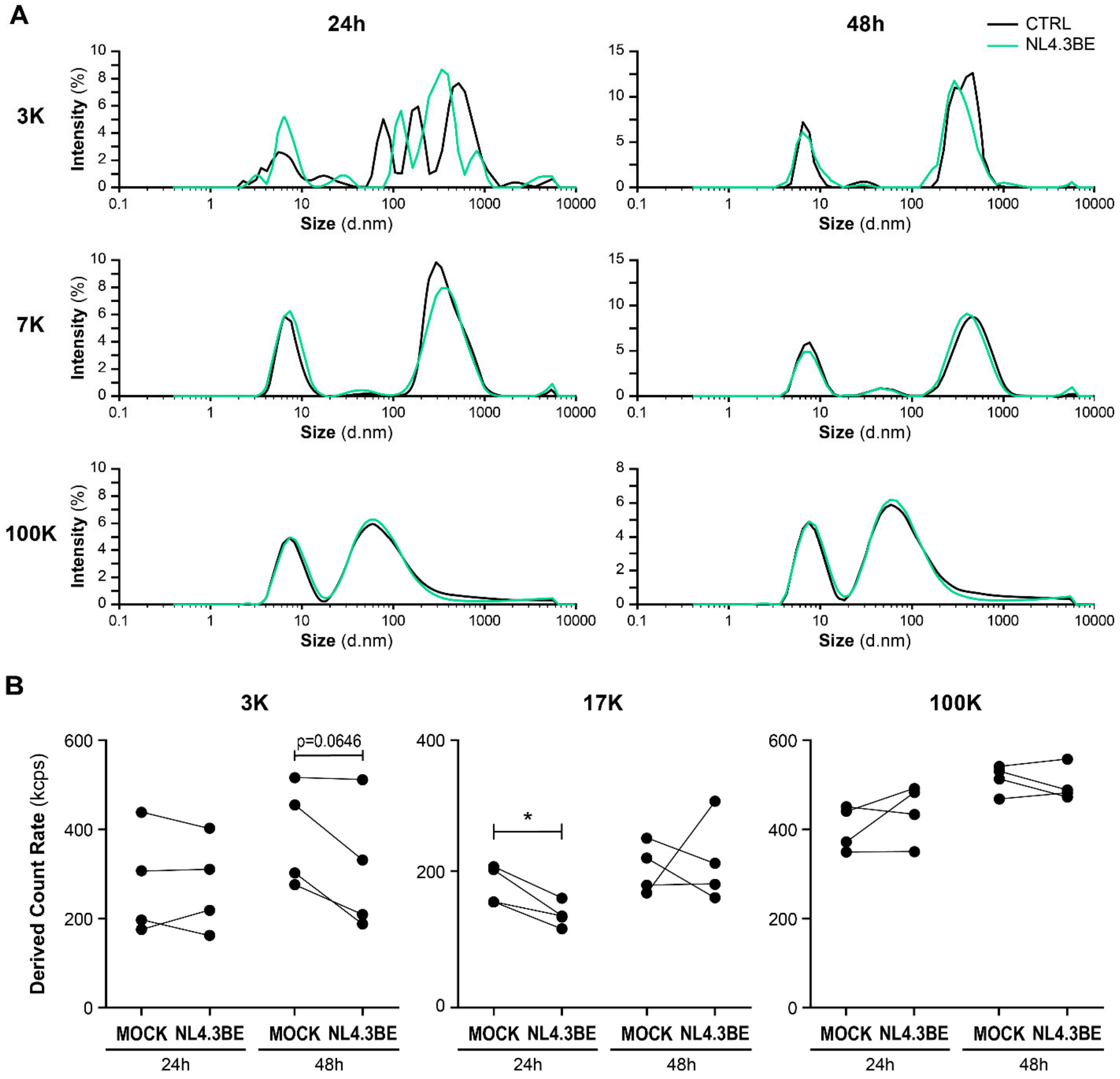

Figure S4. HIV infection has little effect on the size and relative abundance of EVs released by PBMCs

PBMCs freshly isolated from HIV-negative donors (n = 4) were incubated with NL4.3Bal*env* (100 ng of p24 per 10^6 cells) or diluent for 2h, washed three times with PBS to eliminate free virus then incubated for up to 48h. Culture supernatants were incubated with 1 mM AT-2 to inactivate viral particles then centrifuged sequentially at 3,000 × *g* (3K), 17,000 × *g* (17K) and 100,000 × *g* (100K). EV size **(A)** and relative abundance **(B)** were measured by DLS in duplicate at RT with a Zetasizer Nano ZS instrument. Each dot represents one donor. Significant difference (*$p < 0.05$) is based on parametric paired t-tests.

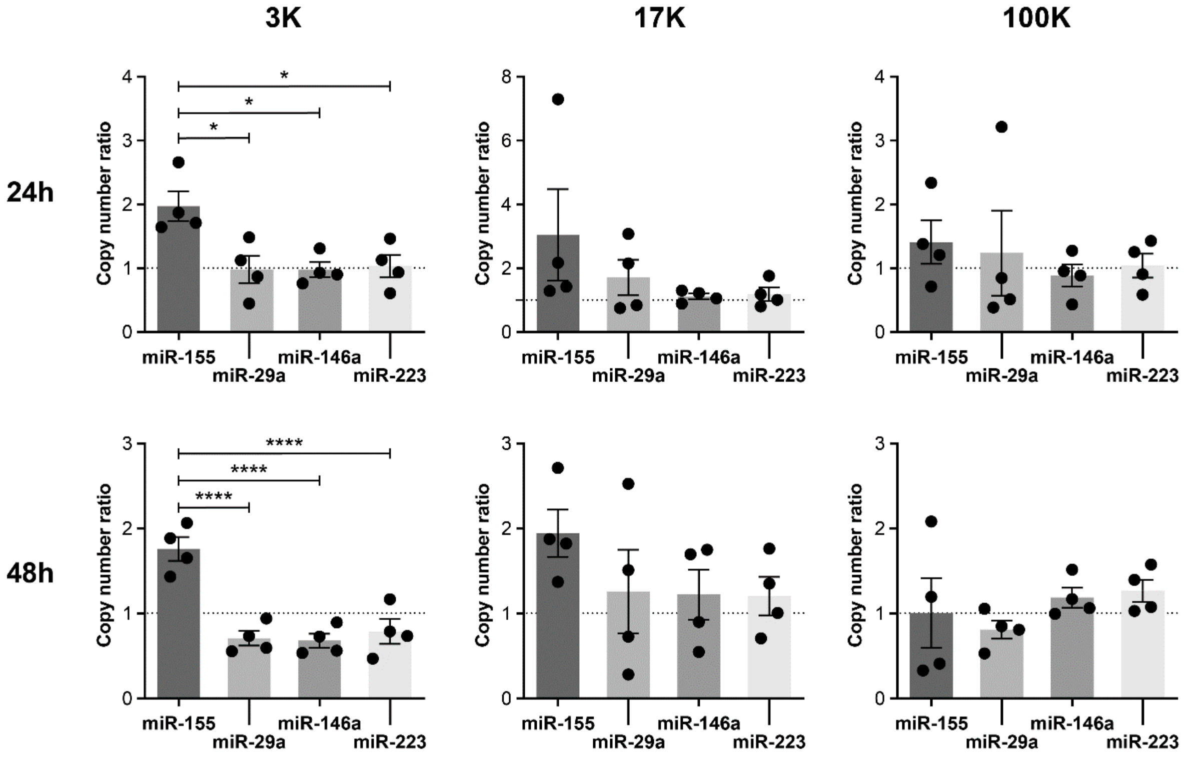

Figure S5. MiR-155 is preferentially enriched in EVs secreted by HIV-infected PBMCs. PBMCs freshly isolated from HIV-negative donors (n = 4) were incubated with NL4.3Bal*env* (100 ng of p24 per 10^6 cells) or diluent for 2h, washed three times with PBS to eliminate free virus then incubated for up to 48h. Culture supernatants were incubated with 1 mM AT-2 to inactivate viral particles then centrifuged sequentially at 3,000 × *g* (3K), 17,000 × *g* (17K) and 100,000 × *g* (100K). Mature miRNA in EVs was quantified by RT-qPCR using a standard curve produced from synthetic miRNA. The copy number ratio is the number of miRNA copies in the NL4.3Bal*env* condition divided by the number in the control condition, which is represented on the graphs by the dotted line (y = 1). Each dot represents one donor. Significant difference (*p < 0.05; ****p < 0.0001) is based on two-way ANOVA with Tukey's multiple comparisons test.

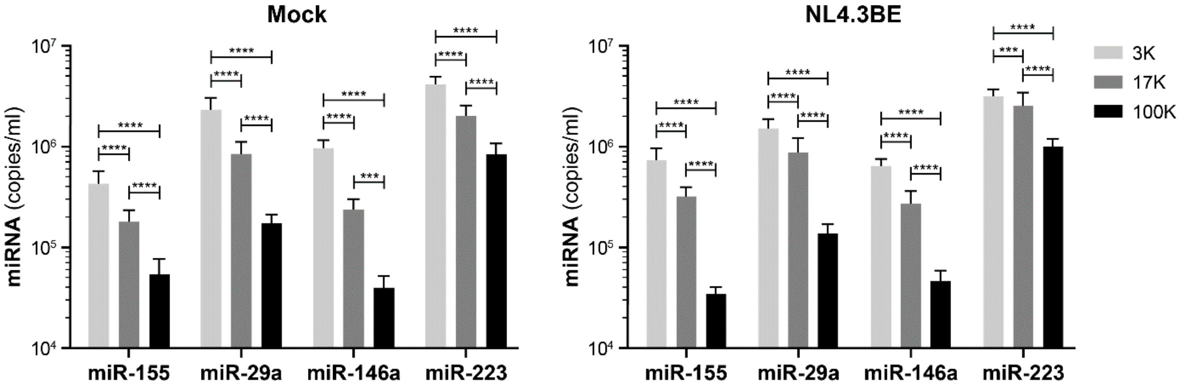

Figure S6. EV-borne miRNAs are associated mostly with larger EVs.
PBMCs freshly isolated from HIV-negative donors (n = 4) were incubated with NL4.3Bal*env* (100 ng of p24 per 10^6 cells) or diluent for 2h, washed three times with PBS to eliminate free virus then incubated for up to 48h. Culture supernatants were incubated with 1 mM AT-2 to inactivate viral particles then centrifuged sequentially at 3,000 × *g* (3K), 17,000 × *g* (17K) and 100,000 × *g* (100K) Mature miRNA in EVs was quantified by RT-qPCR using a standard curve produced from synthetic miRNA. Significant differences (***p < 0.001; ****p < 0.0001) are based on two-way ANOVA with Tukey's multiple comparisons test.

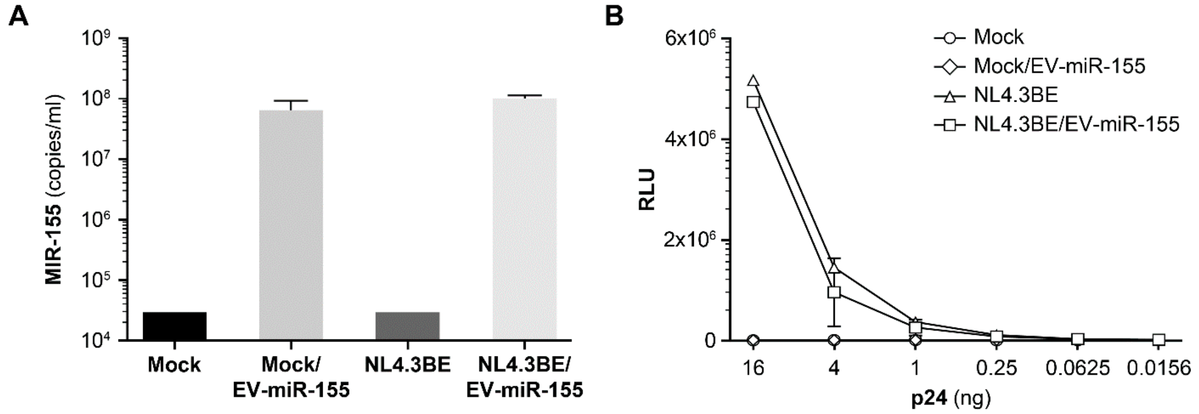

Figure S7. NL4.3BE and EV-miR-155 co-production by HEK293T cells.
HEK293T cells were transfected with pUC.19 (mock treatment) or pNL4.3Bal*Env* (HIV-1 strain NL4.3BE) or co-transfected by adding pMIG-miR-155 (NL4.3BE/EV-miR-155). Virus and EVs were concentrated by centrifuging culture supernatant at 100,000 × *g*. MiR-155 was quantified by RT-qPCR **(A)**. Viral infectivity was measured on TZM-bl cells **(B)**. Plotted values are averages of two experiments (n = 2).

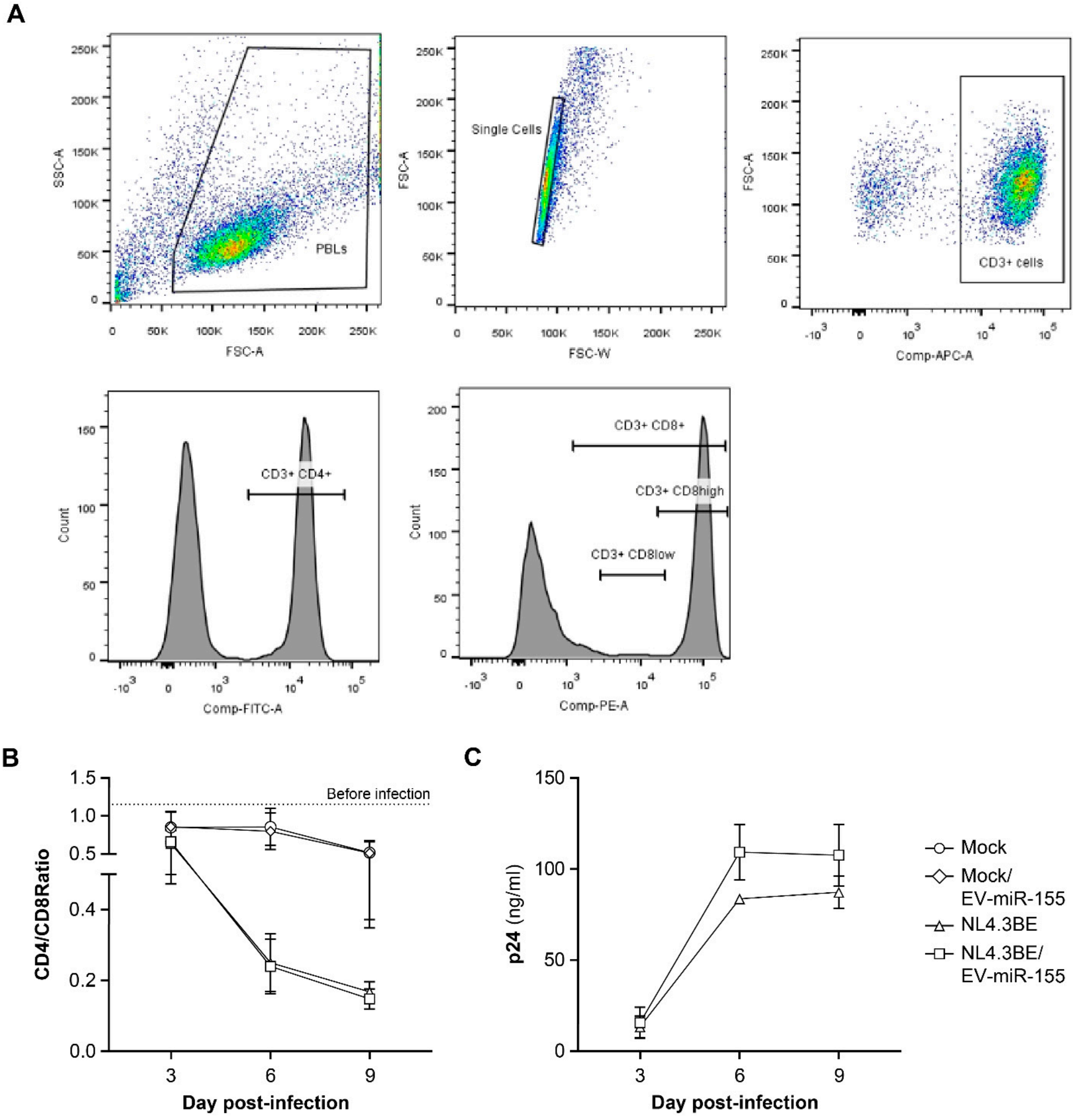

Figure S8. Impact of EV-miR-155 on infection of PBL cells by HIV-1.

PBLs from different donors were contacted with NL4.3BE or NL4.3BE/EV-miR-155 or a mock mixture and cultured for up to 9 days. CD4+ / CD8+ T cell ratio was determined from percentages measured by flow cytometry **(A and B)**. Viral production **(C)** was evaluated by quantitative ELISA against capsid protein p24 (at least two experiments). Plotted values are averages among independent donors (n = 4). Two-way ANOVA with Bonferroni post-test showed no significant differences.

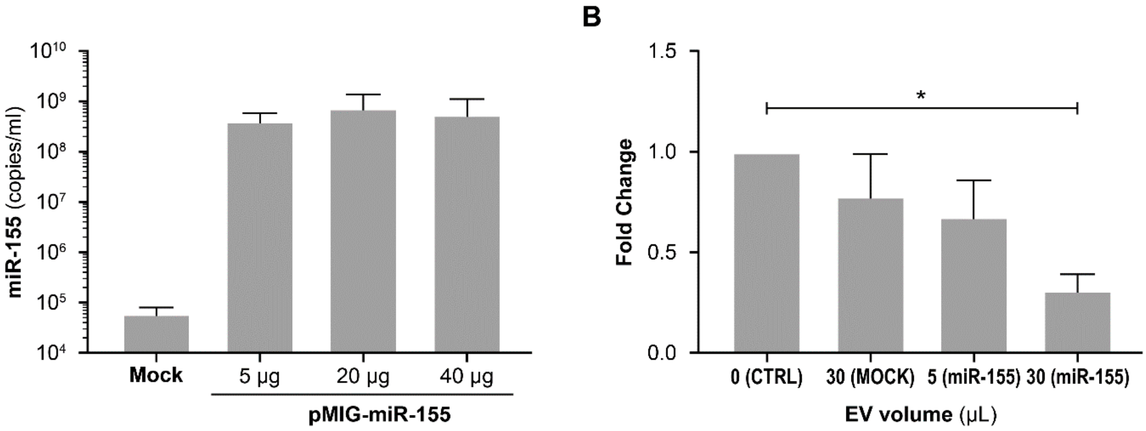

Figure S9. EV-miR-155 downregulates SOCS1 mRNA independently of the infection status. HEK293T cells (n = 2 lots) were transfected using the calcium/phosphate method with 5, 20, or 40 µg of pMIG-miR-155 plasmid or mock-transfected. Culture supernatant was centrifuged at 100,000 × *g*. MiR-155 expression in EVs **(A)** was measured by qRT-PCR. A mix of both EV-miR-155 production was used to stimulate PBMCs (n = 3 donors) for 48h versus EVs from non-transfected HEK293T cells (Mock) **(B)**. Expression of the known miR-155 target SOCS1 was measured by qRT-PCR and normalized to tubulin as housekeeping gene. Relative expression was calculated as $2^{-\Delta\Delta CT}$. Significant difference (*p < 0.05, **p < 0.01, p < 0.001) was based on one-way ANOVA.

	IgG Panel			
Fluorescent dye	*Antigen*	*Isotype*	*Clone*	*Volume / tube (µL)*
FITC	/			5
APC	/			5
PE	/			5

	**Positive Panel Panel 1 : CD4/CD8**			
Fluorescent dye	*Antigen*	*Isotype*	*Clone*	*Volume / tube (µL)*
FITC	**CD4**		OKT4	2.5
APC	**CD3**		SK7	2
PE	**CD8a**		HiT8a	5

Table S1. Antibody panels used for flow cytometry analysis

Target	Primer or probe	Sequence 5’ → 3’	Annealing temperature (°C)
HIV RNA	HIV F	GCCTCAATAAAGCTTGCCTTGA	60
	HIV R	GGCGCCACTGCTAGAGATTTT	
Total HIV DNA (preamplification)	ULF1	ATGCCACGTAAGCGAAACTCTGGGTCTCTC TDGTTAGAC	55
	UR1	CCATCTCTCTCCTTCTAGC	
Integrated DNA (preamplification)	ULF1	ATGCCACGTAAGCGAAACTCTGGGTCTCTC TDGTTAGAC	55
	Alu1	TCCCAGCTACTGGGGAGGCTGAGG	
	Alu2	GCCTCCCAAAGTGCTGGGATTACAG	
Total and integrated DNA (qPCR)	Lambda T	ATGCCACGTAAGCGAAACT	60
	UR2	CTGAGGGATCTCTAGTTACC	
	UHIV TaqMan	FAM-CACTCAAGG/ZEN/CAAGCTTTATTGAG GC	
CD3 (preamplification)	HCD3OUT5’	ACTGACATGGAACAGGGGAAG	55
	HCD3OUT3’	CCAGCTCTGAAGTAGGGAACATAT	
CD3 (qPCR)	HCD3IN5’	GGCTATCATTCTTCTTCAAGGT	60
	HCD3IN3’	CCTCTCTTCAGCCATTTAAGTA	
	CD3 TaqMan	FAM-AGCAGAGAA/ZEN/CAGTTAAGAGCCT CCAT	
PPIA	PPIA F	TGAGAACTTCATCCTAAAGCATAC	60
	PPIA R	CATCCAACCACTCAGTCTTG	
SOCS1	SOCS1 F	GAACCTTCCTCCTCTTCCT	60
	SOCS1 R	GTGATGCGCCGGTAATC	
Lamin B1	Lamin B1 F	GGCGAAGATGTGAAGGTTAT	60
	Lamin B1 R	TCCTCCTCTTCTTCAGGTATG	
IL-15	IL-15 F	GTCCGGAGATGCAAGTATTC	60
	IL-15 R	CTCCAGTTCCTCACATTCTTT	
CD4	CD4 F	GTCCCTTTTAGGCACTTGCTTCT	60
	CD4 R	TCTTTCCCTGAGTGGCTGCT	
CD8	CD8 F	CCCTGAGCAACTCCATCATGT	60
	CD8 R	GTGGGCTTCGCTGGCA	
Pri-miR-155	Pri-miR-155 F	GGTAAATAACATCTGACAGCTAATG	60
	Pri-miR-155 R	GCTCGCACAACCGTATTAT	

*F: forward; R: reverse

Table S2. Primer sets used for PCR
